# DSP-0509, a systemically available TLR7 agonist, exhibits combination effect with immune checkpoint blockade by activating anti-tumor immune effects

**DOI:** 10.3389/fimmu.2023.1055671

**Published:** 2023-01-30

**Authors:** Yosuke Ota, Yasuhiro Nagai, Yuko Hirose, Seiji Hori, Erina Koga-Yamakawa, Ken Eguchi, Kentaro Sumida, Masashi Murata, Hiroki Umehara, Setsuko Yamamoto

**Affiliations:** Cancer Research Unit, Sumitomo Pharma Co. Ltd., Osaka, Japan

**Keywords:** TLR7 agonist, DSP-0509, check point blocker, combination, effector memory T cell

## Abstract

TLR7 is an innate immune receptor that recognizes single-stranded RNAs, and its activation leads to anti-tumor immune effects. Although it is the only approved TLR7 agonist in cancer therapy, imiquimod is allowed to be administered with topical formulation. Thus, systemic administrative TLR7 agonist is expected in terms of expanding applicable cancer types. Here, we demonstrated the identification and characterization of DSP-0509 as a novel small-molecule TLR7 agonist. DSP-0509 is designed to have unique physicochemical features that could be administered systemically with a short half-life. DSP-0509 activated bone marrow-derived dendritic cells (BMDCs) and induced inflammatory cytokines including type I interferons. In the LM8 tumor-bearing mouse model, DSP-0509 reduced tumor growth not only in subcutaneous primary lesions but also in lung metastatic lesions. DSP-0509 inhibited tumor growth in several syngeneic tumor-bearing mouse models. We found that the CD8^+^ T cell infiltration of tumor before treatment tended to be positively correlated with anti-tumor efficacy in several mouse tumor models. The combination of DSP-0509 with anti-PD-1 antibody significantly enhanced the tumor growth inhibition compared to each monotherapy in CT26 model mice. In addition, the effector memory T cells were expanded in both the peripheral blood and tumor, and rejection of tumor re-challenge occurred in the combination group. Moreover, synergistic anti-tumor efficacy and effector memory T cell upregulation were also observed for the combination with anti-CTLA-4 antibody. The analysis of the tumor-immune microenvironment by using the nCounter assay revealed that the combination of DSP-0509 with anti-PD-1 antibody enhanced infiltration by multiple immune cells including cytotoxic T cells. In addition, the T cell function pathway and antigen presentation pathway were activated in the combination group. We confirmed that DSP-0509 enhanced the anti-tumor immune effects of anti-PD-1 antibody by inducing type I interferons *via* activation of dendritic cells and even CTLs. In conclusion, we expect that DSP-0509, a new TLR7 agonist that synergistically induces anti-tumor effector memory T cells with immune checkpoint blockers (ICBs) and can be administered systemically, will be used in the treatment of multiple cancers.

## Introduction

Toll like receptors (TLRs) are one class of the pattern-recognition receptors (PRRs) and play critical roles in infectious disease elimination through pathogen-associated molecular pattern (PAMP) recognition leading to activation of innate and subsequently adaptive immunity (1–3). Compared with other TLRs, TLR3, 7, 8, and 9 are located on endosomes (4). Among TLRs, TLR7 is predominantly expressed on the endosomes of plasmacytoid dendritic cells (pDCs), B cells, and monocytes (5, 6) and it is the TLR that recognizes viral single-stranded RNAs and activate innate immunity (7). TLR7 signal activation using MYD88 as an adaptor protein is reported to trigger anti-tumor immunity by stimulating secretion of inflammatory cytokines such as IL-6 and IL-12 in a NF-kB dependent manner, which are downstream transcription factors, leading to the secretion of type-1 interferons *via* IRF7 (8, 9). Imiquimod is the only approved TLR7 agonist indicated for basal cell carcinoma to date (10, 11). Though several TLR7 agonists are currently in clinical trials, most of compounds are developed as an intratumoral strategy of administration to attenuate systemic immune adverse effects (12). However, in terms of expanding application to other cancer types and anti-tumor immunity to metastatic tumors, the development of systemically administered TLR7 agonists is expected (13). Recently, immune checkpoint blockers, including anti-PD-1/PD-L1 antibodies and anti-CTLA-4 antibodies, have been applied clinically in the treatment of several cancer types. Because of its durable anti-tumor effects, immune checkpoint blocker immunotherapy has replaced conventional cancer therapy as the standard therapy for many types of cancer (14). Although the anti-tumor effect of immune checkpoint blockers is considerable, there is still a medical need to improve the efficacy of these blockers in some patients, as they lose their potency in some patients due to the development of tumor resistance to the anti-tumor effect (15–17). Combined therapy with immunotherapeutic agents is considered one way to enhance the effect of these immune checkpoint blockers. TLR7 agonists enhance antigen-presenting function by acting on antigen-presenting cells (APCs) and this activity promises to be additive/synergistic to the activity of concomitant immune checkpoint blockers, including anti-PD-1/PD-L1 and anti-CTLA-4 antibodies currently used in the clinic (18–20), and therefore, we have focused on combinations with immune checkpoint blockers as promising drug discovery targets. Here, we report DSP-0509 as a novel intravenous injectable small molecule TLR7 agonist which is designed to be rapidly excrete from body to reduce side effects as well as strong anti-tumor activity additively or synergistically combined with immune checkpoint blockers. These data support continued clinical evaluation of DSP-0509 as a combination therapy with immune checkpoint blockers.

## Results

### DSP-0509 exhibited TLR7 selective agonistic activity

**Figure 1:**
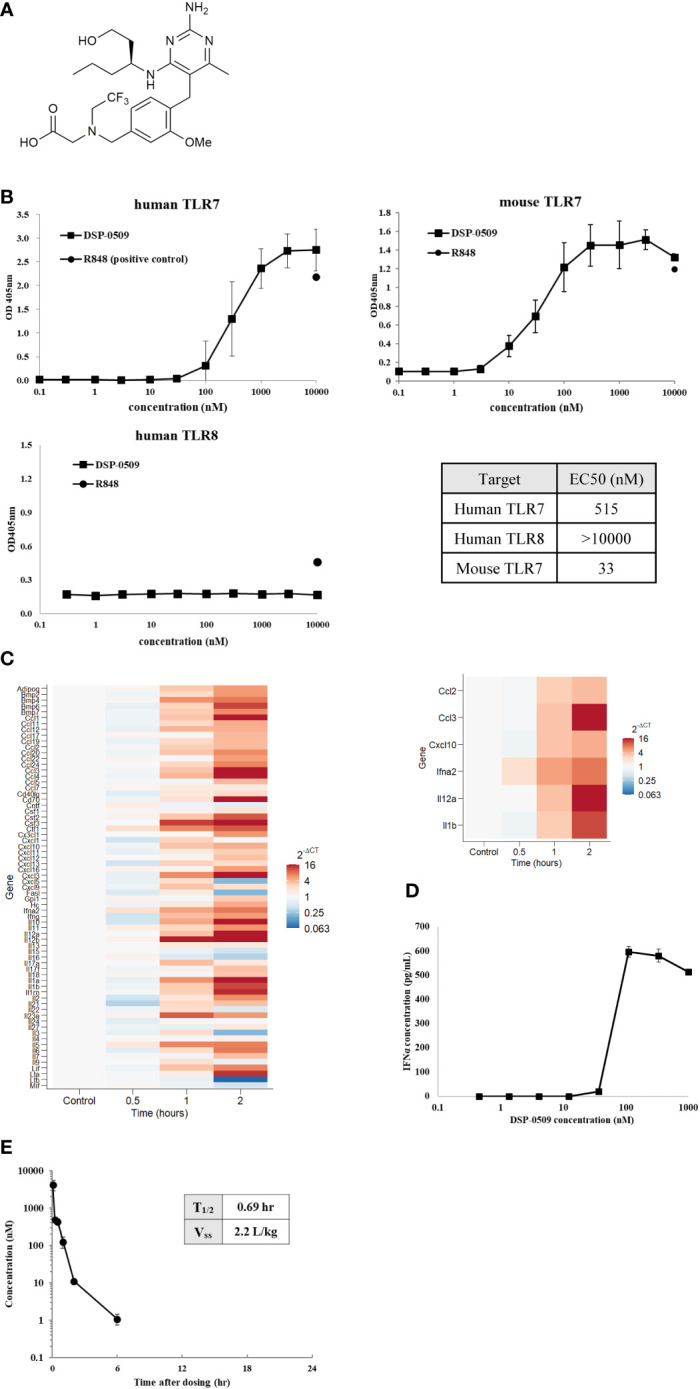
DSP-0509 exhibited TLR7 selective agonistic activity **(A)** Chemical structure of DSP-0509. **(B)** TLR7/NF-κB/SEAP HEK 293 or TLR8/NF-κB/SEAP HEK 293 cells were treated with increasing concentrations of DSP-0509. Fluorescence in the supernatants was evaluated 19-24 h after compound addition. The values are mean ± S.E.M. **(C)** BMDCs were incubated with 1 μM DSP-0509 and collected after 1 h or 2 h and cytokine and chemokine levels were measured by qRT-PCR. **(D)** Human primary pDC were incubated with DSP-0509 for 4 h. IFNα concentration in supernatant was measured by ELISA. The values are the mean ± S.E.M. **(E)** Pharmacokinetics analysis was conducted in the CT26 cell-bearing Balb/c mouse. 5 mg/kg of DSP-0509 was administered intravenously. Blood was collected over time up to 24 h after administration. DSP-0509 concentration was determined by LC-MS/MS.

Here, we investigated DSP-0509 as a new small-molecule TLR7 agonist. The chemical structure of DSP-0509 is shown on **Figure 1A**. DSP-0509 has a pyrimidine scaffold and differs from previously reported TLR7 agonists that have an imidazoquinoline scaffold including imiquimod. When the TLR-agonistic activity of DSP-0509 was examined using NF-kB/SEAP/293 cells stably expressing human TLR7, TLR8, or mouse TLR7, DSP-0509 exhibited agonistic activity in both cells expressing human TLR7 with an EC50 of 515 nM and cells expressing murine TLR7 with an EC50 of 33 nM. It is reported that substrate recognition by TLR8 was similar to substrate recognition by TLR7 (21, 22), and most previously reported TLR7 agonists including R848 had TLR8 agonistic activity as well (23). However, DSP-0509 at up to 10 μM showed no agonistic activity against TLR8 (**Figure 1B**). These results indicated that DSP-0509 exhibited TLR7 selective agonistic activity. Since it has been reported that TLR7 is predominantly expressed in pDCs, we investigated whether DSP-0509 activates bone marrow-derived dendritic cells (BMDCs) differentiated from murine bone marrow cells. Cells were harvested 60 and 120 minutes after the addition of 1 μM DSP-0509 to analyze the expression levels of immune-related mRNA by qPCR. DSP-0509 induced the secretion of cytokines including type-I interferons and the elevation of MHC class II expression. This suggested that DSP-0509 led to the activation of dendritic cells through the TLR7 of BMDCs (**Figure 1C**). Furthermore, we investigated the effect of DSP-0509 in human primary pDC. Cells were harvested 4 h after the addition of sequential dose of DSP-0509. The concentration of IFNα in supernatant was analyzed by ELISA. DSP-0509 induced IFNα dose dependent manner (**Figure 1D**). In order to confirm the pharmacokinetics of DSP-0509, blood concentrations of DSP-0509 over time were analyzed in CT26 tumor-bearing mice after administration of a 5 mg/kg i.v. bolus of the compound. The results showed that the DSP-0509 level in blood decreased to below the lower limit of quantification after 6 h. The body half-life of DSP-0509 was calculated to be 0.69 h, which revealed that it was excreted from the body very rapidly. The relatively low volume of distribution (2.2 L/kg) revealed that DSP-0509 has pharmacokinetic properties with low tissue distribution and rapid excretion from the body (**Figure 1E**).

### DSP-0509 induced systemic cytokine response in TLR7 dependent manner

**Figure 2:**
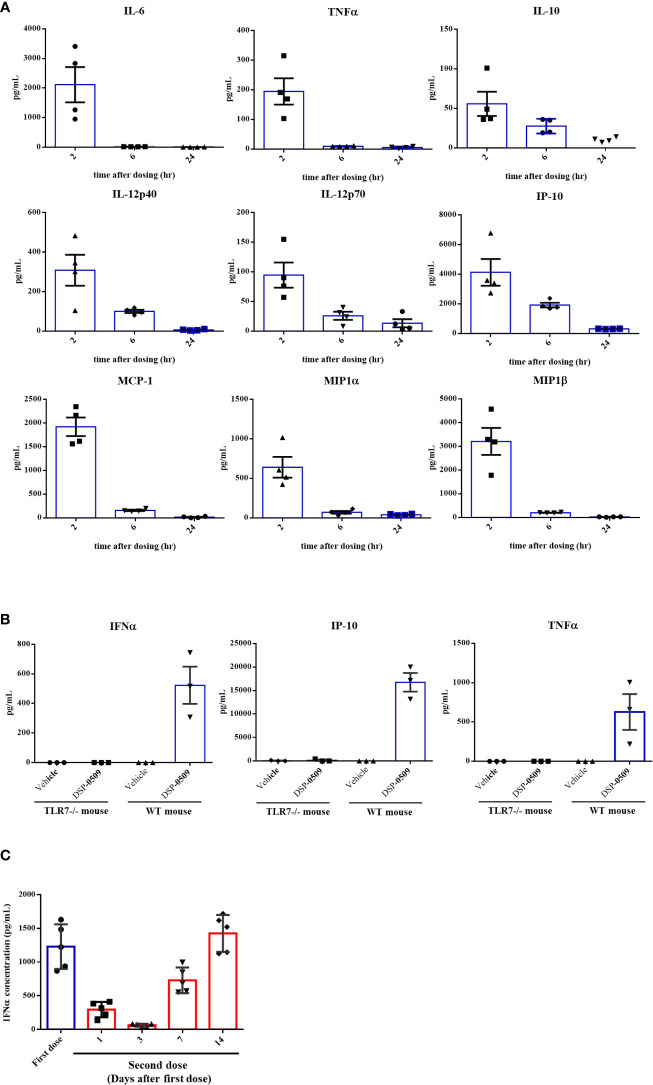
DSP-0509 induced systemic cytokine response in TLR7 dependent manner **(A)** CT26 cell-bearing Balb/c mice were treated with 1 mg/kg of DSP-0509 intravenously. Blood was collected after 2 h, 6 h, and 24 h respectively and the concentration of cytokines were measured by the Luminex assay. The values are the mean of 4 independent studies. **(B)** TLR7 KO mice or wild-type Balb/c mice were treated with 5 mg/kg of DSP-0509 intravenously. Blood was collected after 2 h and the concentration of IFNα was measured by ELISA and other cytokines were measured by the Luminex assay. The values are the mean ± S.E.M. of each group. **(C)** Balb/c mice were treated with 5 mg/kg of DSP-0509 intravenously at day 0 as a first dose and on day 1, 3, 7, or 14 as the second administration, and whole plasma was collected 2 h after the last administration. Plasma IFNα was measured by ELISA. The values are the mean ± S.E.M. of each group (n=5/group).

To assess if DSP-0509 activates TLR7 *in vivo*, cytokines and chemokines were measured in the CT26-bearing mouse model after i.v. bolus administration of 1 mg/kg DSP-0509. All measured cytokine and chemokine levels were increased 2 h after administration and had returned to baseline 24 h after administration (**Figure 2A**). Since IFNα induction has been reported as a hallmark of pDCs activation *in vivo*, we evaluated plasma cytokine concentration after administering a 5 mg/kg i.v. bolus of DSP-0509 to verify whether DSP-0509 exhibits TLR7 agonistic activity *in vivo*. In wild-type mice, marked increases in IFNα, TNFα, and IP-10 concentration were detected in the blood 2 h after DSP-0509 administration, whereas secretions of these cytokines were dramatically attenuated in TLR7 knockout mice (**Figure 2B**). These findings indicated that DSP-0509 activates downstream transcription factors *via* TLR7 in DCs, leading to cytokine-induction both *in vitro* and *in vivo*. It is reported that repeated TLR7 stimulation makes cytokine response weaker due to the tolerance (24). Thus, we assessed IFNα induction after second dosing at 1, 3, 7 and 14 days after the first dose. Interestingly, IFNα induction after second dose was affected by dosing interval. 1 and 3 days interval after first dose was not sufficient for IFNα induction after second dose comparable to first dose (**Figure 2C**). Based on these we applied 7-days interval in the following *in vivo* study. We also evaluated cytokine induction in human whole blood. Human whole blood was incubated for 4 h in the presence of DSP-0509 or R848. Plasma concentration of IFNα2 was higher than that of TNFα or IL-6 in by DSP-0509 treatment. On the other hand, plasma concentration of IFNα2 was lower than that of TNFα or IL-6 in by R848 treatment (**Supplementary Figure 1**). These different cytokine profile would be explained by TLR7 selective profile of DSP-0509.

### T cell-dependent anti-tumor effects were critical for *in vivo* efficacy of DSP-0509

**Figure 3:**
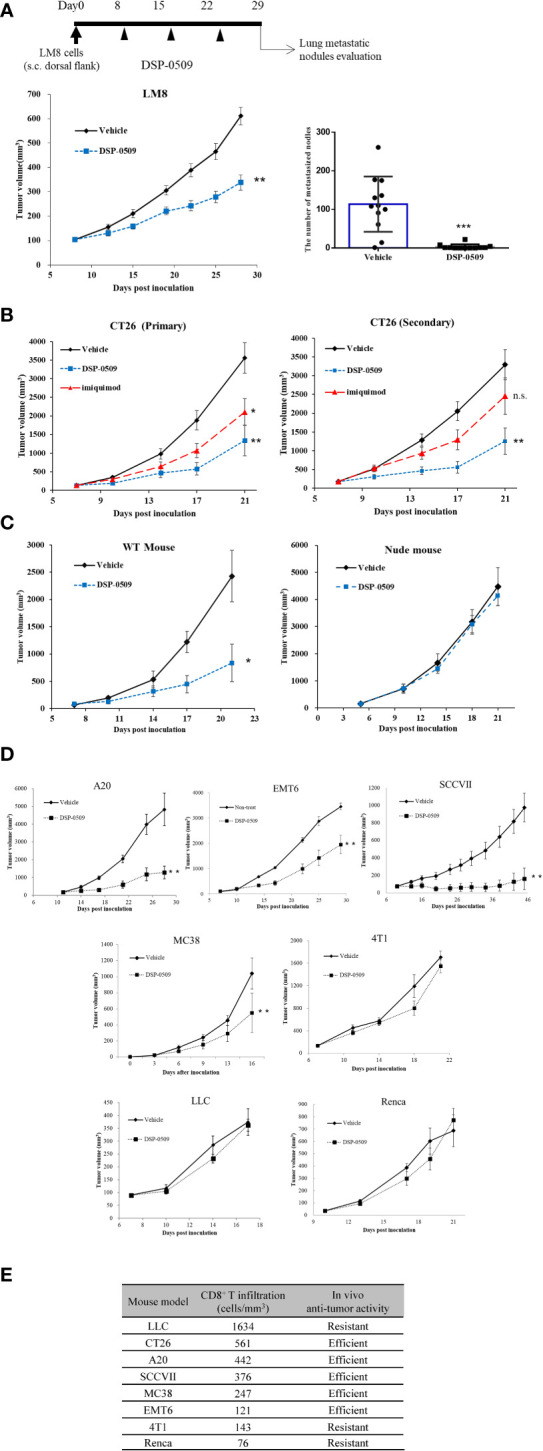
T cell-dependent anti-tumor effects were critical for *in vivo* efficacy of DSP-0509 **(A)** LM8 cells were inoculated in C3H mice. 1 mg/kg of DSP-0509 was administered i.v. once a week from day 8. The tumor volume is the mean ± S.E.M. of each group (n=12/group), and differences were evaluated by the Dunnett test (**P<0.01). The number of metastasized nodes in the lung was evaluated 28 days after inoculation. The values are mean ± S.E.M of each group and differences were determined by the t-test (***P<0.001). **(B)** CT26 cells were inoculated in the subcutaneous both right and left dorsal flanks of Balb/c mice. 5 mg/kg of DSP-0509 was administered i.v. once a week from Day 7. 5% imiquimod cream was applied to primary tumor 4 times a week. The values are the mean ± S.E.M. of each group (n=6/group), and differences were determined by the Dunnett test (*P<0.05) (**P<0.01). **(C)** CT26 cells were inoculated in the subcutaneous dorsal flanks of Balb/c mice and Balb/c nude mice. 5 mg/kg of DSP-0509 was administered i.v. once a week from Day 5. The values are the mean ± S.E.M. of each group (n=6/group), and differences were determined by the Dunnett test (*P<0.01). **(D)** Each cell line was inoculated in the subcutaneous dorsal flanks of the respective host mouse. 5 mg/kg of DSP-0509 was administered i.v. once a week. The tumor volume is the mean ± S.E.M. of each group, and differences were evaluated by the Dunnett test (**P<0.01) **(E)** The frequency of CD8^+^ T cell at the tumor site in each model was analyzed by flow cytometry. Tumors were collected on days 5-10 after inoculation. Efficient in the graph means DSP-0509 reduced tumor growth statistically significant in **Figure 3D**. ns, not significant.

As a next step, we evaluated the anti-tumor effects of DSP-0509 in syngeneic tumor-bearing mouse models as DSP-0509 was shown to have TLR7 agonistic activity and to activate the immune system *in vivo*. In a tumor-bearing mouse model subcutaneously implanted with LM8 mouse osteosarcoma cells, tumor growth was significantly suppressed in the group that received weekly i.v. bolus administration of DSP-0509 1 mg/kg compared with the vehicle treatment group (**Figure 3A**). Since LM8 cells are known to metastasize to the lung after subcutaneous transplantation (25), the effect of DSP-0509 on lung metastasis was evaluated by counting the number of nodules metastasized to the lung. The number of pulmonary metastatic nodules in the DSP-0509 treatment group was significantly lower than that in the vehicle treatment group (**Figure 3A**). To determine whether i.v. administration of DSP-0509 has superior anti-tumor activity against distal tumor compared to topical use of TLR7 agonist, CT26 cells were injected at both dorsal flanks, defined as “primary” and “secondary” tumor. Intravenous dosing of DSP-0509 reduced tumor growth in both primary and secondary tumors. On the other hand, applying imiquimod cream to primary tumor showed anti-tumor activity only in primary tumors (**Figure 3B**). These results indicated that i.v. administration of DSP-0509 inhibited not only the growth of the primary tumor but also its metastasis. To evaluate whether DSP-0509 directly induce immunogenicity in tumor cells, we evaluated the expression of PD-L1 and H2Kd on CT26 cells in the presence of DSP-0509. The expression of PD-L1 and H2Kd on CT26 was not increased by DSP-0509. On the other hand, the expression of PD-L1 and H2Kd was increased in the conditioned medium which is derived from spleen cells added with DSP-0509 (**Supplementary Figure 2**). These data indicated that DSP-0509 had no direct immunogenic or cytotoxic activity on tumor cells, but induced immunogenicity *via* immune cells. In the murine colorectal CT26 tumor-bearing mouse model, weekly i.v. bolus treatment with DSP-0509 5 mg/kg compared with control treatment resulted in significant tumor growth inhibition (**Figure 3C**). To clarify which immune cells contribute to this anti-tumor activity, DSP-0509 was evaluated in nude mice engrafted with CT26 cancer cells. DSP-0509 in these mice showed no clear tumor growth inhibitory activity (**Figure 3C**). Nude mice are T-cell deficient because they are athymic, suggesting that DSP-0509 shows T-cell-dependent tumor growth inhibition in CT26 models. To investigate whether the T cell-dependent anti-tumor effects identified in the CT26 model are applicable to other tumor-bearing models, we evaluated the tumor growth inhibitory activity of DSP-0509 in seven tumor-bearing mouse models. When given weekly, i.v. bolus DSP-0509 5 mg/kg had a significant tumor growth inhibitory effect in the A20, MC38, SCCVII, and EMT6 groups compared with the control group. On the other hand, there was no tumor-growth inhibitory effect in the Renca, 4T1, and LLC models (**Figure 3D**). To identify factors contributing to the differences in anti-tumor activity between these models, we isolated tumor-infiltrating lymphocytes (TILs) from each model at pre-treatment and analyzed CD8^+^ T cells by flow cytometry. The CD8^+^ T cell ratios in CD45^+^ cells from each tumor bearing model is summarized in **Figure 3E**. We found that anti-tumor activity of DSP-0509 tended to be stronger in the tumor-bearing mouse model with high CD8^+^ T cell infiltration and weaker in the tumor-bearing mouse model with low CD8^+^ T cell infiltration. These results suggested that infiltration of the tumor with CD8^+^ T cells at pre-treatment is one of the predictive factors for anti-tumor activity of DSP-0509.

### DSP-0509 in combination with anti-PD-1 antibody additively enhanced anti-tumor activity

**Figure 4:**
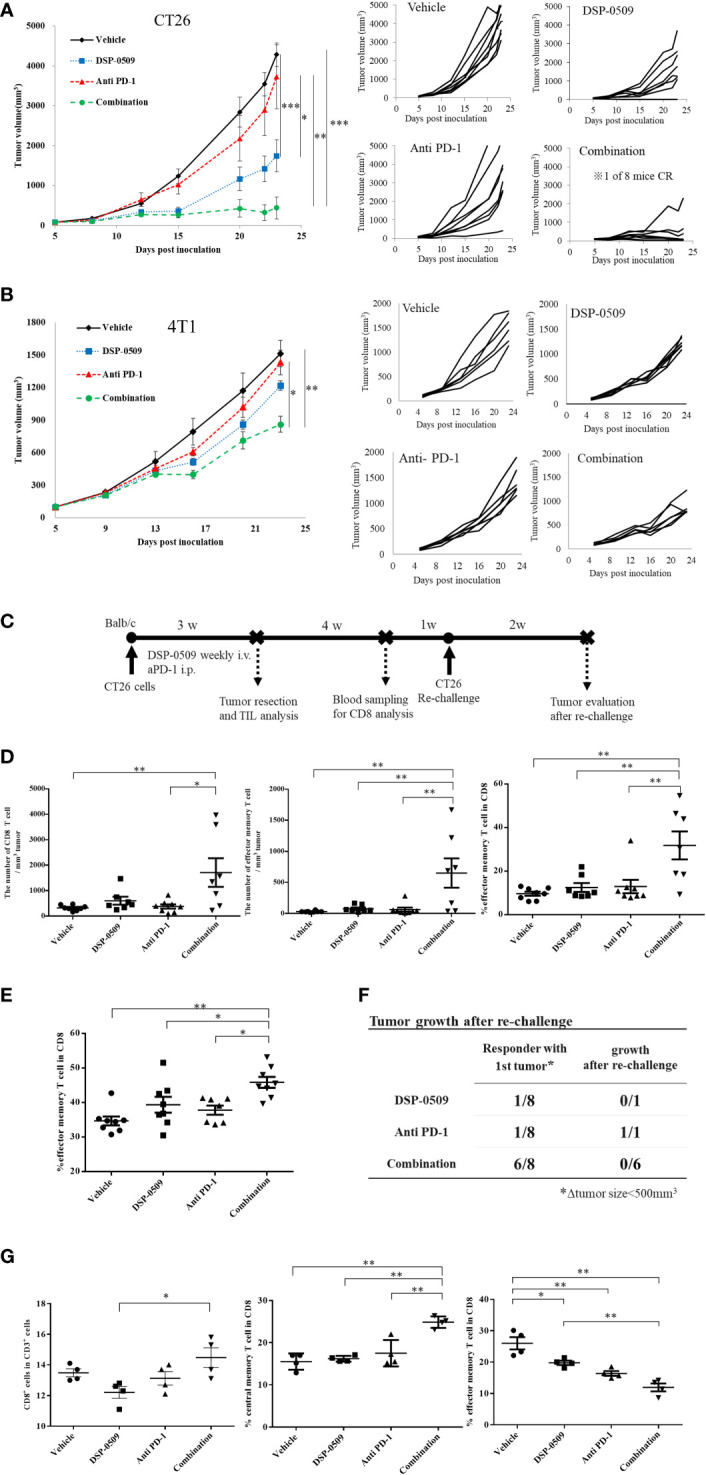
DSP-0509 in combination with anti-PD-1 antibody additively enhanced anti-tumor activity **(A)** CT26 tumor was inoculated subcutaneously into the dorsal flanks of Balb/c mice on day 0. 5 mg/kg of DSP-0509 was administered intravenously on days 5, 12, and 19. 200 µg/mouse of anti-PD-1 antibody (RMP1-14) was administered intraperitoneally on days 5, 8, 12, 15, and 19. The tumor volume is the mean ± S.E.M. of each group (n=8/group). Differences in the day-23 tumor volume were determined by the Tukey test (*P<0.05, **P<0.01, ***P<0.001). **(B)** The 4T1 tumor was inoculated subcutaneously into the dorsal flanks of Balb/c mice on day 0. 5 mg/kg of DSP-0509 was administered intravenously on days 6, 13, and 20. 200 µg/mouse of anti-PD-1 antibody (RMP1-14) was administered intraperitoneally on days 6, 9, 13, 16, and 20. Tumor volume is the mean ± S.E.M of each group (n=6/group). Differences in the day-23 tumor volume were determined by the Tukey test (*P<0.01, **P<0.001). **(C)** Study schedule. **(D)** The number of CD8^+^ T cells and effector memory T cells in TILs was analyzed flow cytometry. The frequency of effector memory T cells the CD8^+^ T cell population was analyzed by flow cytometry. The effector memory T cells were defined as CD62L^-^CD127^+^ cells in the CD8^+^ T cell gate. The value is the mean ± S.E.M. Differences were determined by the Tukey multiple comparison tests (*P<0.05, **P<0.01). **(E)** The frequency of effector memory T cells the CD8^+^ T cell population in peripheral blood lymphocytes was analyzed by flow cytometry. The effector memory T cells were defined as CD62L^-^CD127^+^ cells in the CD8^+^ T cell gate. The value is the mean ± S.E.M. Differences were determined by the Tukey multiple comparison tests (*P<0.05, **P<0.01) **(F)** Tumors that responded to treatment after 3 weeks (were less than 500 mm^3^ in diameter) were resected. Tumor-resected mice were rechallenged with CT26 cells after a recovery period of 5 weeks. The number of mice with tumor growth 2 weeks after rechallenge was assessed. **(G)** Lymph node profiling by flow cytometry. Lymph node was collected 2 h after second dose of DSP-0509. The percentage of central memory T cells and effector memory T cell in CD8^+^ T cells were defined as CD62L^+^CD44^+^ and CD62L^-^CD44^+^ respectively. The value is the mean ± S.E.M. Differences were determined by the Tukey multiple comparison tests (*P<0.05, **P<0.01).

Since the anti-tumor activity of DSP-0509 was dependent on CD8^+^ T cells, we next verified whether DSP-0509 combined with anti-PD-1 antibody enhances anti-tumor activity. In the subcutaneously injected CT26 mouse model, significant tumor growth inhibition was obtained after once-weekly administration of DSP-0509 (5 mg/kg i.v.) but not after twice-weekly administration of the anti-PD-1 antibody (200 μg i.p.). On the other hand, combining both treatments further enhanced the anti-tumor activity of each monotherapy alone (**Figure 4A**). In the combination group, one of eight mice showed complete tumor regression. When the combination of DSP-0509 with anti-PD-1 antibody was also examined in the 4T1 subcutaneous transplantation model, significant tumor growth inhibition was observed in the combination group but not in each monotherapy group (**Figure 4B**). We did not find obvious body weight reduction in both CT26 and 4T1 models (**Supplementary Figure 3**). Since the anti-tumor activity was enhanced by the combination of DSP-0509 with anti-PD-1 antibody in the CT26 model, we analyzed CD8^+^ T cells in TILs as well as in peripheral blood (**Figure 4C**). Tumors were collected after 3-week treatment with each compound or the combination and assessed the number of CD8^+^ T cells and effector memory T cells (CD62L^-^CD127^+^ cells) in TILs by flow cytometry. Both the number of CD8^+^ T cells and effector memory T cells were increased in the combination group (**Figure 4D**). Furthermore, we analyzed the effector memory CD8^+^ T cell ratio in CD8^+^ T cells. We found that the percentage of effector memory T cells in the CD8^+^ T cells was significantly increased in the combination treatment group compared to the vehicle and monotherapy groups, respectively (**Figure 4D**). In addition, we surgically resected tumors from mice that responded to treatment (defined as mice having tumor volumes less than 500 mm^3^ after 3-week treatment). When we evaluated peripheral blood by flow cytometry 3 weeks after tumor resection, we found that the percentage of effector memory T cells (CD62L^-^CD127^+^ cells) in the CD8^+^ T cells in peripheral blood was significantly increased in the combination treatment group compared to the vehicle and monotherapy groups, respectively (**Figure 4E**). Furthermore, we re-implanted CT26 cells to tumor resected mice and assessed tumor regrowth for 2 weeks and observed no tumor regrowth in all cases in the combination group (**Figure 4F**). We also assessed another population in PBMC. The percentage of Treg in CD4^+^ T cells (Foxp3^+^CD4^+^ cells) was not changed in any groups but the percentage of DC in CD3^-^ cells (CD11c^+^CD3^-^) was decreased in all groups compared to vehicle group (**Supplementary Figure 5**). In addition, lymph node analysis showed that the percentage of CD8^+^ T cells in CD3^+^ cells was increased in combination group compared to DSP-0509 group. Interestingly, the percentage of central memory T cells (CD62L^+^CD44^+^ cells) in CD8^+^ T cells was increased in the combination group compared to the vehicle and each monotherapy groups. We also found that the percentage of effector memory T cells (CD62L^-^CD44^+^ cells) in CD8^+^ T cells was decreased in all group compared to vehicle group (**Figure 4G**). The percentage of Treg was not changed in all group (**Supplementary Figure 5**). These results suggested that the combination of DSP-0509 with anti-PD-1 antibody significantly enhanced tumor growth inhibition in both the CT26 and 4T1 models. We showed that combination of DSP-0509 with anti-PD-1 antibody enhanced the infiltration of CD8^+^ T cells in tumor. The immune memory effect preventing the regrowth of re-implanted tumor in the combination treatment group can be partly explained by the increasing of effector memory T cell induction.

### DSP-0509 in combination with anti-CTLA-4 antibody significantly enhanced anti-tumor activity

**Figure 5:**
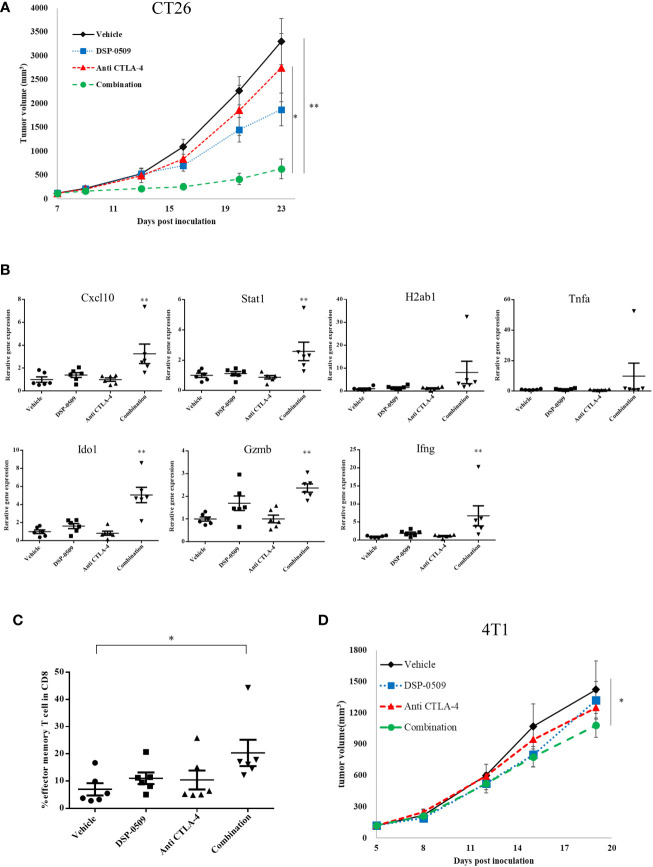
DSP-0509 in combination with anti-CTLA-4 antibody significantly enhanced anti-tumor activity **(A)** CT26 tumor was inoculated subcutaneously into the dorsal flanks of Balb/c mice on day 0. 5 mg/kg of DSP-0509 was administered intravenously on days 7, 14, and 21. 200 μg/mouse of anti-CTLA4 antibody (9F10) was administered intraperitoneally on days 7, 9, 14, 16, and 20. Tumor volume is the mean ± S.E.M. of each group (n=6/group). Differences in day-23 tumor volume were determined by the Tukey test (*P<0.05, **P<0.01). **(B)** mRNA was isolated from the tumor on day 26. Expression data were normalized to Gapdh expression and are shown as the mean ± S.E.M. of each group (n=6/group). Differences were determined by the Dunnett test (**P<0.01). **(C)** TILs were isolated from tumors on day 26. The frequency of effector memory T cells in the CD8^+^ T cell population was analyzed by flow cytometry. Effector memory T cells were defined as CD62L^-^CD127^+^ cells in the CD8^+^ T gate. The value is the mean ± S.E.M. Differences were determined by the Tukey multiple comparison test (*P<0.05). **(D)** 4T1 tumors were inoculated subcutaneously into the dorsal flanks of Balb/c mice on day 0. 5 mg/kg of DSP-0509 was administered intravenously on days 5, 12, and 21. 200 μg/mouse of anti-CTLA4 antibody (9F10) was administered intraperitoneally on days 5, 8, 12, 15, and 19. Tumor volume is the mean ± S.E.M of each group (n=6/group). Differences in the day-19 tumor volume were determined by the Dunnett test (*P<0.05).

Since the combination of DSP-0509 and anti-PD-1 antibody enhanced anti-tumor activity, we next evaluated the anti-tumor activity of the combination with anti-CTLA-4 antibody, a different immune-checkpoint blocker from the anti-PD-1 antibody used clinically. CT26 subcutaneous inoculation model mice treated with DSP-0509 5 mg/kg i.v. weekly combined with anti-CTLA-4 antibody 200 μg i.p. twice weekly showed significant tumor growth inhibitory activity compared to the vehicle and anti-CTLA-4 antibody treated groups (**Figure 5A**). In addition, following tumor resection on day 26 and RNA extraction, the immune response in the tumor was examined by analyzing the IFNγ-related gene (26) using qRT-PCR. We found that Cxcl10, Stat1, Ido1, Gzmb, and Ifnγ expressions were increased in the combination treatment group compared to the vehicle group. The expression of H2ab1 and Tnfa tended to be increased in the combination group, but no statistical differences were observed (**Figure 5B**). Furthermore, we isolated TILs on day 26 from tumors and analyzed the effector memory T cells (CD62L^-^CD127^+^ cells) in the CD8^+^ T cells by flow cytometry and found that effector memory T cell infiltration was significantly enhanced in the combination treatment group compared to the vehicle treatment group (**Figure 5C**). We also evaluated absolute number of CD8^+^ T cells and effector memory T cells. These cell number tended to be increased in combination group but the difference was not statistically significant (**Supplementary Figure 6**). To investigate whether the combination of DSP-0509 and anti-CTLA-4 antibody enhances tumor growth inhibitory effects in other models, the combination was also investigated in 4T1 model mice. Although not significant in each monotherapy group, there was a significant tumor growth inhibitory effect in the combination group compared to the vehicle group (**Figure 5D**). Thus, we confirmed that DSP-0509 additively elevated tumor growth inhibition even in combination with anti-CTLA-4 antibody. These anti-tumor activities were accompanied by activation of TILs in the tumor microenvironment and increased effector memory T cell number.

### IFNγ-related genes were upregulated by DSR-0509 combined with anti-PD-1 antibody

**Figure 6:**
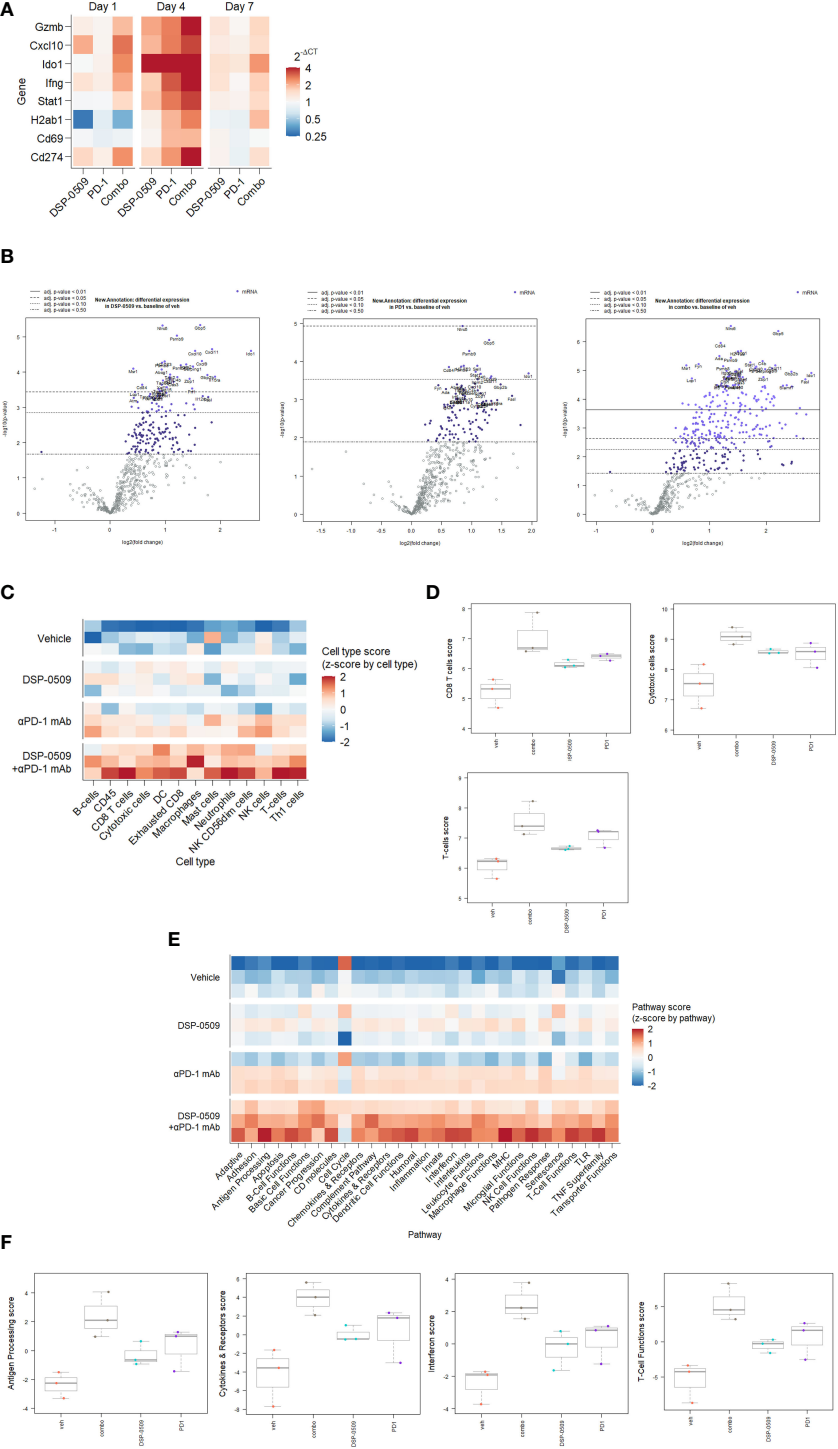
IFNγ-related genes were upregulated by DSR-0509 combined with anti-PD-1 antibody **(A)** When the tumor volume of CT26-bearing mice reached around 100 mm^3^, DSP-0509 5 mg/kg i.v. and/or anti-PD-1 antibody (RMP1-14) 200 μg/mouse i.p. were administered. Tumor samples for qRT-PCR analysis were collected on day 1, 4, or 7 after dosing, respectively. The gene expression value is the mean of 3 mice/group. **(B)** Volcano plots of nCounter mRNA expression data were drawn by the nSolver Advanced Analysis software package. **(C, D)** Cell type profiling was conducted using the nSolver Advanced Analysis software package (n=3/group). **(E, F)** Pathway score analysis was conducted using the nSolver Advanced Analysis software package (n=3/group).

Since the combination of DSP-0509 with anti-PD-1 antibody synergistically enhanced tumor growth inhibitory activity, gene expression analysis was performed using RNA extracted from the CT26 tumors from the implanted mouse model to evaluate the immune response comprehensively in the tumor microenvironment. IFNγ-related genes expression was analyzed by qRT-PCR in tumor samples collected at 1, 4, and 7 days after initial administration of DSP-0509 or anti-PD-1 antibody. We found transient upregulation of IFNγ-related genes that reached a peak at 4 days after administration in all treatment groups. Upregulation was prolonged particularly in the combination treated group, but returned to baseline in the DSP-0509 and anti-PD-1 antibody monotherapy groups (**Figure 6A**). Thus, we found in the tumor microenvironment that immune activation by the combination of DSP-0509 with anti-PD-1 antibody was most remarkable at 4 days after treatment. Next, to analyze tumor immune environment comprehensively, we performed nCounter analysis using mRNA derived from whole tumor collected at 4 days after the initial administration of DSP-0509, anti-PD-1 antibody, or the combination. Analysis of immune-related mRNA expression was performed by using a mouse pan-cancer immune profiling panel with the nCounter system. Volcano plots (**Figure 6B**) show the change of gene expression in all groups compared to the vehicle group. The rate of significantly changed gene expression for DSP-0509, anti-PD-1 antibody, and the combination was 38.4%, 34.9%, and 55.7%, respectively (**Figure 6B**). Among the genes with changed expression, Fasl and Ido1 showed highly increased expression in the combination group, suggesting that effector cells including CTLs were activated, and the IFNγ receptor pathway in tumors was activated in response to the effector cell stimulation. Cell type analysis was performed using the nSolver application and found that DSP-0509 and anti-PD-1 antibody monotherapy increased the scores of T cells, CD8^+^ T cells, and cytotoxic cells and combination treatment further increased these scores compared to each monotherapy (**Figures 6C**, **D**). Most of the cell type scores except for NK cells were increased in combination group (**Supplementary Figure 7**). These results indicated that the combination of DSP-0509 with anti-PD-1 antibody further increased the infiltration or growth of immune cells in the tumor microenvironment compared to each monotherapy. In addition, pathway score analysis was performed using mRNA expression data analyzed by the nSolver application. Pathway scores, except the cell cycle score, tended to increase in the combination group compared with the monotherapy groups (**Figures 6E, F**). In particular, the antigen processing score, cytokine and receptors score, interferon score, and T cell function score were significantly higher in the combination group than in each monotherapy group. Most of other pathway scores were increased in combination group (**Supplementary Figure 8**). These findings indicated that the peak of immune activation in the tumor microenvironment stimulated by the combination of DSP-0509 with anti-PD-1 antibody was reached 4 days after administration, and that the combination therapy dramatically enhanced anti-tumor immune activity.

## Materials and methods

### Compounds and dosing solutions

DSP-0509 and R848 were chemically synthesized at Sumitomo Pharma., Co Ltd. Detailed chemical synthesis was described in the patent of WO/2013/172479. For *in vitro* studies, 10 mM solutions were prepared by dissolution in DMSO followed by dilution of the DMSO up to a final concentration of 0.1%. For *in vivo* studies, the compounds were dissolved in 2.5 mM glycine buffered solution of pH 10.2. 5% imiquimod cream was purchased from Mochida Pharmaceutical Co., Ltd (Beselna Cream 5%).

### Cells and cultures

CT26, 4T1, EMT6, LL2, and A20 were obtained from the American Type Culture Collection (ATCC, Manassas, VA). SCCVII was kindly provided by the group studying particle beam therapy of tumors at the Kyoto University Reactor Research Institute. MC38 was kindly provided by Dr. Kawakami, Keio University. Renca cells were kindly provided by Dr. Fujioka, Iwate Medical University School of Medicine. LLC cells were obtained from RIKEN BRC. CT26, 4T1, and Renca cells were maintained in culture by passage 1-2 times a week in RPMI1640 supplemented with 10% FCS, penicillin/streptomycin. LLC cells were maintained by sub-culturing cells 1-2 times per week in DMEM supplemented with 10% FCS, penicillin/streptomycin. SCCVII cells were maintained by sub-culturing cells 1-2 times per week in MEM supplemented with 10% FCS and penicillin/streptomycin.

### Mice

We used 6- to 10-week-old Balb/c or Balb/c nu/nu mice purchased from Charles River Japan or CLEA Japan, 6- to 10-week-old C57/BL6 mice purchased from Charles River Japan, 6- to 10-week-old C3H/HeN mice purchased from Charles River Japan, and TLR7 KO (Balb/c) mice purchased from Oriental Biodevices. All animal studies were conducted in compliance with the Sumitomo Pharma Animal Ethics Code.

### *In vivo* anti-tumor study

CT26, 4T1, EMT6, A20, and Renca cells were suspended in HBSS and implanted subcutaneously into Balb/c mice. LM8 and SCCVII cells were implanted into C3H mice. LLC and MC38 were implanted into C57/BL6 mice. The number of cells for implantation was 1 × 10^6^ cells for CT26, 4T1, EMT6, A20, LLC, and MC38, 1 × 10^5^ cells for Renca, 2.5 × 10^6^ cells for LM8, and 5 × 10^6^ cells for SCCVII. When the tumors reached approximately 100 mm^3^, the mice were randomly divided into groups. Bolus i.v. DSP-0509 was administered at 5 mg/kg in all studies except for the LM8 model mouse study in which bolus i.v. DSP-0509 was given at 1 mg/kg, and each dose was administered once a week until the end of the study. Anti-PD-1 antibody (BioXcell, RMP1-14) and anti-CTLA-4 antibody (BioXcell, 9H10) were administered twice weekly at 200 μg intraperitoneally in all studies. Dosing was started on the same day as the start of DSP-0509. The tumor volume was calculated using the formula (L × W^2^)/2, where L and W refer to the length and width dimensions, respectively. All *in vivo* studies are repeated at least 2 times independently.

### TLR7 and TLR8 *in vitro* reporter assays

The TLR7/NF-κB/SEAP HEK 293 cell line was obtained from Discovery BioScience. The TLR8/NF-κB/SEAPorter HEK 293 cell line was obtained from IMGENEX. All cell lines were maintained as instructed by the vendor. To assess TLR agonistic activity, the cell suspension was diluted with cell culture medium to a viable cell density of 22 × 10^4^ cells/mL, then the cells were seeded into a 96-well plate at 90 μL/well, and the plate was incubated in a CO_2_ incubator for 2-5 h. After incubation, 10 μL of compound in medium was added. The 96-well plate was then placed in a CO_2_ incubator, and the cells were incubated for 19-24 h. The cells were incubated at room temperature for about 10 min after the addition of 50 μL of pNPP solution. The reaction in each well was stopped by addition of 50 μL of 4M NaOH, and the absorbance of the chromogenic product was measured at 405 nm using a SpectraMax190 (Molecular Devices) or ELX808 (BioTek) plate reader. EC50 was calculated by Stat Preclinica Client (SAS 9.2, Takumi Information Technology Inc.)

### Bone marrow-derived dendritic cells and pDC assay

Bone marrow cells were collected from Balb/c mice and used for BMDC differentiation as previously reported (27). Briefly, bone marrow cells were harvested from Balb/c mice by flushing both femora with PBS. To harvested cells seeded into 6-well plates, we added recombinant mouse GM-CSF 20 ng/mL, changed the medium after 3 days, and finally cultured the cells for 6 days. The adherent cells were used as BMDCs. Compounds were added to this cell and 1 or 2 h later cells were harvested and RNAs were extracted and used for qRT-PCR. Primary human BDCA4^+^ (ALLCELLS) cells was used as pDC. To access IFNα secretion, pDCs of 2 × 10^4^ cells suspended in RPMI1640 supplemented with 10% FBS, penicillin/streptomycin was seeded in round bottom 96-well plate. After serial concentration of DSP-0509 was added, pDCs were cultured for 4h in 37°C, 5% CO2 incubator. IFNα concentration in culture supernatant was measured by human IFN-alpha multi-subtype ELISA (PBL).

### qRT-PCR analysis

Total RNA was collected from *in vivo* implanted tumors that were snap frozen on dry ice. After homogenization of the tumors, a Maxwell^®^ RSC simplyRNA Tissue Kit (Promega) was used with the Maxwell^®^ RSC Instrument (Promega) to isolate total RNA. The resulting total RNA was subjected to reverse-transcription-PCR using a SuperScript VILO cDNA Synthesis kit (Invitrogen) to synthesize cDNA. The synthesized cDNA was used in a qRT-PCR analysis according to a TaqMan gene expression assay protocol. The probes for the TaqMan gene expression assay used in this study were as follows: Mm99999915 g1 Gapdh, Mm00445235 m1 Cxcl10, Mm00442837 m1 Gzmb, Mm00443258 m1 Tnf, Mm01168134 m1 Ifng, Mm01257286 m1 Stat1, Mm00492590 m1 Ido1, and Mm00439216 m1 H2-Ab1. mRNA expression analysis in BMDCs was performed using a RT² Profiler™ PCR Array System Kit for Mouse Cytokines & Chemokines (QIAGEN). Gene list included in the panel and raw data is shown in **Supplementary Table 1**. A CFX384 (Bio-Rad) or ABI 7900 (Applied Biosystems) instrument was used for the quantitative real time PCR detection.

### Pharmacokinetics study

CT26-bearing BALB/c mice were administered a single intravenous dose (5 mg/kg), and blood samples for determination of the plasma concentrations were collected at 5, 15, 30 minutes, 1, 2, 6, and 24 h after administration. Plasma concentrations of DSP-0509 were determined using an LC-MS/MS setup (LC: Shimadzu LC-10AD, LC-20AD [SHIMADZU CORPORATION]; MS/MS: API-4000, QTRAP-5500 [AB Sciex]).

### Cytokine measurement

Whole blood samples were obtained 2 h after intravenous administration of DSP-0509 at 5 mg/kg to Balb/c and TLR7KO mice, respectively. The plasma was separated from the collected blood after centrifugation. The cytokine levels were measured using a mouse IFNα ELISA kit (PBL Biomedical Laboratories), Milliplex mouse cytokine/chemokine panel (Merck Millipore) for the various other cytokines according to the manufacturer’s instructions. For human cytokine measurement, IFNα multi-subtype ELISA kit (PBL Biomedical Laboratories), Milliplex human cytokine/chemokine panel (Merck Millipore) for the IFNα2, TNFα and IL-6 according to the manufacturer’s instructions. Luminex 200 (Luminex) and Elx808 (BioTek) analyzers were used for detection of each assay.

### Flow cytometry analysis

Tumors excised from mice immediately after euthanasia were used for isolation of tumor-infiltrating lymphocytes. After cutting the tissue into 2-3 mm^3^ squares, single cells were acquired from the pieces using a mouse tumor dissociation kit (Miltenyi Biotech) and gentleMACS™ Octo Dissociator (Miltenyi Biotech). Additionally, TILs were isolated by treatment with ACK buffer to remove the erythrocytes after density-gradient centrifugation using Percoll (GE Healthcare). Flow cytometry analysis was carried out using the following antibodies: FITC-CD8a (BD 53-6.7), PerCP-Cy5.5-CD62L (eBioscience, MEL-14), PE-Cy7-CD127 (eBioscience, A7R34), FITC-CD44 (BD Pharmingen, IM7), PE-CD62L (BD Pharmingen, MEL-14), PerCP CD8a (Biolegend, 53-6.7), APC-Foxp3 (Miltenyi, 3G3), PE-Cy7-CD3e (BD Pharmingen, 145-2C11), FITC-CD3e (Biolegend, 17A2), PE-Cy7-CD11c (BD bioscience, HL3), BV510-CD4 (Biolegend, GK1.5), Fixable Viability Stain 450 (BD Horizon) and Fixable Near-IR Dead Cell Stain (Invitrogen). Foxp3/Transcription Factor Staining Buffer (eBioscience) was used for intracellular staining. To analyze flow data, Flow Jo (BD Biosciences) was used. In **Figure 4C**, the effector memory T cell population was analyzed by gating the CD62L^-^CD127^+^ population of CD8^+^ T cells after gating out FVS 450-positive dead cells and gating the lymphocyte population in the FSC/SCC plot (**Supplementary Figure 4**). In **Figure 4G**, the central memory T cell and the effector memory T cell population was analyzed by gating the CD62L^+^ CD44^+^ in CD8^+^ T cells and CD62L^-^CD44^+^ in CD8^+^ T cells respectively (**Supplementary Figure 4**).

### nCounter analysis

After tissue homogenization, RNA was extracted using a Maxwell^®^ RSC Instrument (Promega). In the nCounter analysis, a PanCancer Immune Profiling Panel (NanoString Technologies) was used to perform gene-expression analysis according to the manufacturer’s protocol. The obtained data were analyzed using nSolver 4.0 software (NanoString) and nSolver advanced analysis software (NanoString) for cell type profiling and measuring pathway scores. The list of 770 genes included in the panel and the normalized raw data of mRNA expression were described in **Supplementary Table 2**.

### Statistical analysis

Stat Preclinica Client (SAS 9.4, Takumi Information Technology Inc.) was used for statistical analysis in the *in vivo* anti-tumor studies. nSolver advanced analysis software (NanoString) was used for nCounter analysis. To compare the two groups data, unpaired two tailed t-test was conducted. *In vivo* tumor study, one-way repeated ANOVA was used and followed by Dunnet test or Tukey test was used as *post hoc* analysis. Data are presented as mean ± SEM.

## Discussion

The typical TLR7 agonists previously reported, including Imiquimod, have a structure based on the imidazoquinoline scaffold, whereas DSP-0509 has its own unique structure, which is based on the pyrimidine scaffold. Although DSP-0509 has no agonistic effect on TLR8, many TLR7 agonists with an imidazoquinoline skeleton also have agonistic effects on TLR8, suggesting that the pyrimidine backbone of DSP-0509 may contribute to its TLR7 selectivity (**Figure 1B**). Since the activating effect of TLR7 on pDCs is stronger than that of TLR8 (28), treatment with TLR7-selective DSP-0509 may pose less of a risk of systemic immune toxicity, because the induction of inflammatory cytokines, including TNFα, by DSP-0509 may be lower than that of TLR8 selective agonists. In fact, we clearly showed that DSP-0509 less induced TNFα compared to R848 of TLR7/8 dual agonist (**Supplementary Figure 1**). DSP-0509 administered i.v. had a half-life of less than 1 h and was confirmed to be rapidly excreted (**Figure 1E**). DSP-0509 is designed to have a short half-life in the body when intravenously administered to prevent excessive immune activation leading to systemic inflammatory side effect, which was the drawback of using other TLR7 agonists (29–31). In addition, the higher clearance of the drug may also be explained by organic anion transporting poly peptide (OATP), which is a DSP-0509 efflux transporter in the kidney (data not shown). Although DSP-0509 induced various inflammatory cytokine in mouse, the cytokine level is expected to be lower than that induced by R848 based on previous report (32). By balancing immunostimulatory and systemic immune side effects, DSP-0509 may possess physicochemical properties that overcome the limitations of existing TLR7 agonists. In addition, we clearly showed superior metastatic inhibitory activity of DSP-0509 compared to the that of topical treatment of imiquimod. (**Figure 3B**). We think that the metastasis inhibitory effect observed here can be one of the advantages of intravenously administering TLR7 agonists. Currently, several TLR7 agonists are being developed to reduce systemic side effects by intratumoral administration. Although intratumoral administration of the drug is expected to have anti-tumor immune activity on untreated tumors due to an abscopal effect, the effect is considered to be limited (33). On the other hand, systemic immune activation in addition to immune activation in tumor microenvironment has been reported to be critical for effective anti-tumor immune activation (34), suggesting that the potent inhibition of metastatic tumor growth by intravenous administration of DSP-0509 effectively induces systemic immune activation. We showed that anti-tumor activity of DSP-0509 was dependent on CD8^+^ T cell in tumor (**Figure 3C**). Since it is reported that the anti-tumor effect of the TLR7 agonist imiquimod is CD8^+^ T cell dependent (35), we showed that DSP-0509 has the same mechanism of anti-tumor activity. Since the anti-tumor effect of DSP-0509 was found to be T-cell dependent in the CT26 model, we analyzed the invasion of CD8^+^ T cells in pre-treatment tumors and found a tendency toward higher percentage of CD8^+^ T cells in the model in which the anti-tumor effect was observed (**Figure 3D**). However, despite the high infiltration of CD8^+^ T cells in the LLC model, DSP-0509 did not have anti-tumor activity in this model. This may be caused by lower cell-surface MHC class I expression (36). And although the infiltration of CD8^+^ T cells was low in the EMT6 model, DSP-0509 had anti-tumor effects. This may be due to mediation by immune cells other than CD8^+^ T cells. The activation of myeloid cells and NK cells in addition to the activation of cytotoxic T cells was reported after TLR7 agonist stimulation (37, 38), and the increased invasion of myeloid cells was reported in EMT6 model mice (39), suggesting immune cells other than CD8^+^ T cells contribute to the anti-tumor effects of DSP-0509 in the EMT6 model. Although several mechanisms of anti-PD-1 antibody resistance have been reported, the reported mechanisms may be overcome by combination therapy with TLR7 agonists. The mechanisms include failure of antigen presentation, failure of immune cells to infiltrate the tumor, failure of the interferon-gamma pathway in the tumor, and the contribution of immune suppressive cells (40). Thus, the combination of DSP-0509 with anti-PD-1 antibody may lead to the enhancement of the anti-tumor immune effects of anti-PD-1 antibody and even overcome resistance. In fact, the combination of DSP-0509 with anti-PD-1 antibody had an enhanced tumor-growth inhibitory effect in CT26 model mice (**Figure 4A**). In this model, a significant tumor growth inhibitory effect was observed with DSP-0509 alone, but not anti-PD-1 antibody alone. The anti-tumor activity of DSP-0509 was further enhanced by combination with the anti-PD-1 antibody, and tumor regression was complete in some animals. Therefore, it is considered that the effect of the combination was synergistic. Furthermore, we found no body weight (**Supplementary Figure 3**.) loss in this study, suggesting that DSP-0509 combined with anti-PD-1 antibody may pose less of a risk of systemic immune toxicity. When the combination of DSP-0509 and anti-PD-1 antibody was also investigated in 4T1 model mice, none of the single agents had a significant tumor growth inhibitory effect, but the combination treatment did when compared to the vehicle treatment (**Figure 4B**). Previous reports have shown that in the 4T1 tumor-bearing mouse model there were fewer CD8^+^ T cells infiltration and more infiltration of immunosuppressive cells including MDSCs. Therefore, it is known that the tumors in the 4T1 model have an immune-excluded microenvironment (41). Our data may suggest that the combination of DSP-0509 and anti-PD-1 antibody additively activates anti-tumor immune effects and leads to anti-tumor effects even in an immunosuppressive microenvironment. In CT26 model mice, the effector memory CD8^+^ T cells in TILs and peripheral blood were significantly increased by treatment with the combination of DSP-0509 with anti-PD-1 antibody (**Figures 4D and 4E**). It has previously been reported that effector memory CD8^+^ T cells are more abundant in tumors that have responded to anti-PD-1 antibody (42). We also found that the population of central memory T cell in lymph node was increased in combination group and effector memory T cell in lymph node was decreased in combination group (**Figure 4G**). Considering the lymph node and tumor analysis, we speculate that the combination of DSP-0509 with anti-PD-1 antibody induced central memory T cell in lymph node and differentiation from central memory T cell to effector memory T cell in tumor. Memory T cell formulation is not fully understood but some differentiation model has been proposed so far (43). To induce memory T cells efficiently, IL-7 and IL-15 is reported to be important role (44, 45). Interestingly, DSP-0509 induced IL-7 in BMDC (**Figure 1C**) and IL-15 was increased in combination group in CT26 nanostring data. We think upregulation of these cytokines can induce formation of central memory T in lymph node and effector memory cells in tumor. It has also been reported that *de novo* methylation of memory-related genes inhibits differentiation into effector memory T cells when CD8^+^ T cells cannot be activated by anti-PD-1 antibody (46). Taken together with our findings, these studies indicate that the combination of DSP-0509 with anti-PD-1 antibody may have induced the demethylation of memory-related genes in CD8^+^ T cells in the tumor and induced their differentiation into effector memory T cells, but further investigation will be needed. The combination of DSP-0509 with anti-PD-1 antibody induced complete tumor rejection after rechallenge with tumor cells (**Figure 4F**). Observed significant tumor rejection can be explained by effector memory induction. Since tumor rejection was observed in the mouse after tumor resection, the combination of DSP-0509 with anti-PD-1 antibody may be used in a neoadjuvant setting in clinical trials. DSP-0509 also showed enhanced anti-tumor activity in combination with anti-CTLA-4 antibody (**Figure 5A**, **Figure 5D**). It is known that CTLA-4 is mainly expressed on CTLs (47) and Tregs (48). Since CTLA-4 on CTLs is known to inhibit CD28 signal by binding CD80/86 (49) and CTLA-4 on Treg is reported to be essential molecule for immune suppressive activity of Treg (50). Therefore anti-CTLA-4 antibody is used for cancer immunotherapy (51). To enhance anti-tumor activity, TLR agonists was combined with anti-CTLA-4 antibody both in preclinical study and clinical trial (52, 53). Based on these backgrounds, it is conceivable that the combination of anti-CTLA-4 antibody with DSP-0509 activated CTLs in the tumor microenvironment *via* activation of CTLs and Treg depletion. From the results of the analysis of gene expression in the tumor, Ifnγ and Gzmb were elevated in the combination group, suggesting that CTLs were activated in the tumor. The increased expression of Stat1, Ido1, and H2ab1 suggested that the IFNγ signal in the tumor was upregulated and accompanied by elevated IFNγ expression in CTLs (**Figure 5B**). We also found that the combination of DSP-0509 with anti-CTLA-4 antibody resulted in increased effector memory T cells in the tumor (**Figure 5C**). Since the intensity of TCR stimulation on CTLs has been reported to be critical in inducing differentiation of memory T cells (54), it is conceivable that the combination of DSP-0509 with anti-CTLA-4 antibody increased the antigen-presenting ability of DCs, resulting in increased TCR signal on CTL. Recent approach in TLR7 agonist drug discovery is focusing on tumor targeting modality including ADC and nanoparticle and so on (55–58). However, these approaches are not always succeeded so far. We think activating both systemic and tumor site is important for achieving optimal anti-cancer immunity, and we believe DSP-0509 would be a possible solution for this approach. In this study, we clarified that DSP-0509 activated the innate immune system *via* TLR7, which in turn resulted in activation of the adaptive immune system, and that it showed anti-tumor activity. It became clear that the combination with the immune checkpoint blockers had strong anti-tumor effect. Currently, DSP-0509 is in the efficacy testing stage of clinical trials (NCT03416335), and future trial results are expected.

## Data availability statement

The original contributions presented in the study are publicly available. This data can be found here: https://www.ncbi.nlm.nih.gov/geo/ under the accession number GSE216890.

## Ethics statement

The studies involving human participants were reviewed and approved by Sumitomo Pharma Ethical Committee. The patients/participants provided their written informed consent to participate in this study.

## Author contributions

YO is main corresponding author. All authors contributed to the article and approved the submitted version.
