# Supplementary material for: DSP-0509, a systemically available TLR7 agonist, exhibits combination effect with immune checkpoint blockade by activating anti-tumor immune effects
Source: Front Immunol. 2023 Jan 30;14:1055671. doi: 10.3389/fimmu.2023.1055671 (PMC9922899; doi:10.3389/fimmu.2023.1055671)
Supplement: Supplementary file 1 [file DataSheet_1.docx]

Supplementary Material

# Supplementary Figures and Tables

## Supplementary Figure 1.


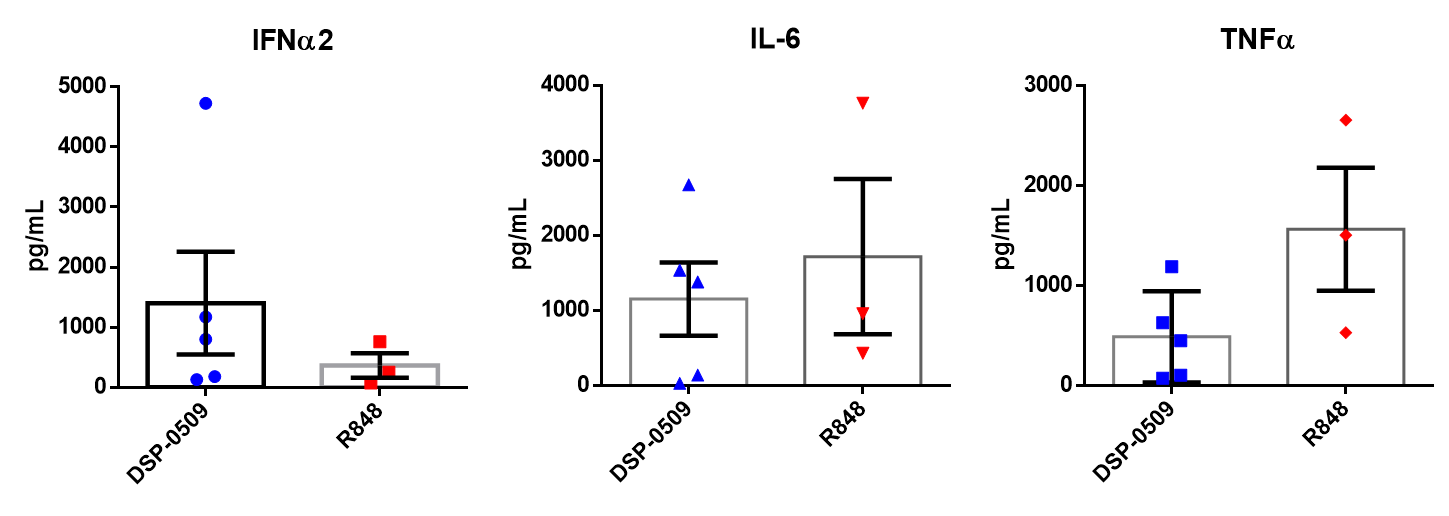


**Supplementary Figure 1.** Cytokine induction in whole blood of healthy volunteer. Human whole blood was incubated for 4 h in the presence of 1 μM of R848 or DSP-0509. Plasma cytokine concentration was measured by multiplex immune assay.

## Supplementary Figures 2.


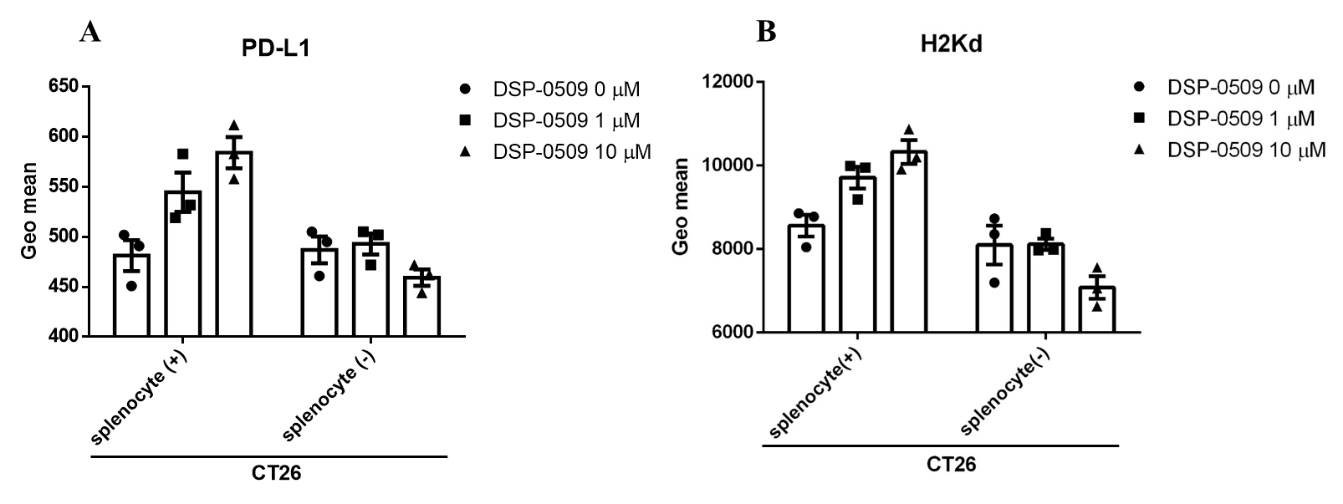


**Supplementary Figure 2.** **A.** PD-L1 expression on CT26 was analyzed by flow cytometry in the presence or absence of splenocytes treated with DSP-0509. Splenocytes were treated with each concentration of DSP-0509. Conditioned medium was added to CT26. Flow cytometry analysis was evaluated after 16 h culture. **B.** H2Kd expression on CT26 was analyzed by flow cytometry in the presence or absence of splenocytes treated with DSP-0509. Splenocytes were treated with each concentration of DSP-0509. Conditioned medium was added to CT26.

## Supplementary Figures 3.


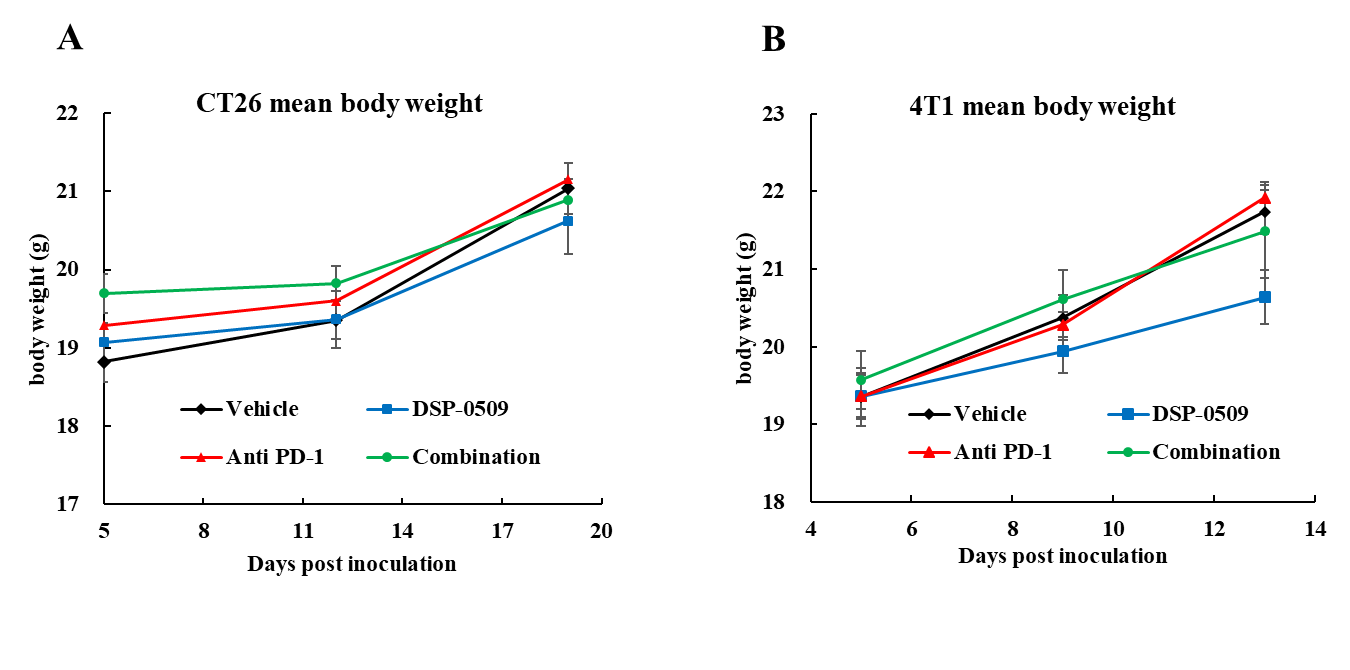


**Supplementary Figure 3.** **A.** Body weight change in CT26 model. Mice were treated with DSP-0509 and/or anit-PD-1 antibody as shown in Fig. 4A. **B.** Body weight change in 4T1 model. Mice were treated with DSP-0509 and/or anit-PD-1 antibody as shown in Fig. 4B.

## Supplementary Figure 4.


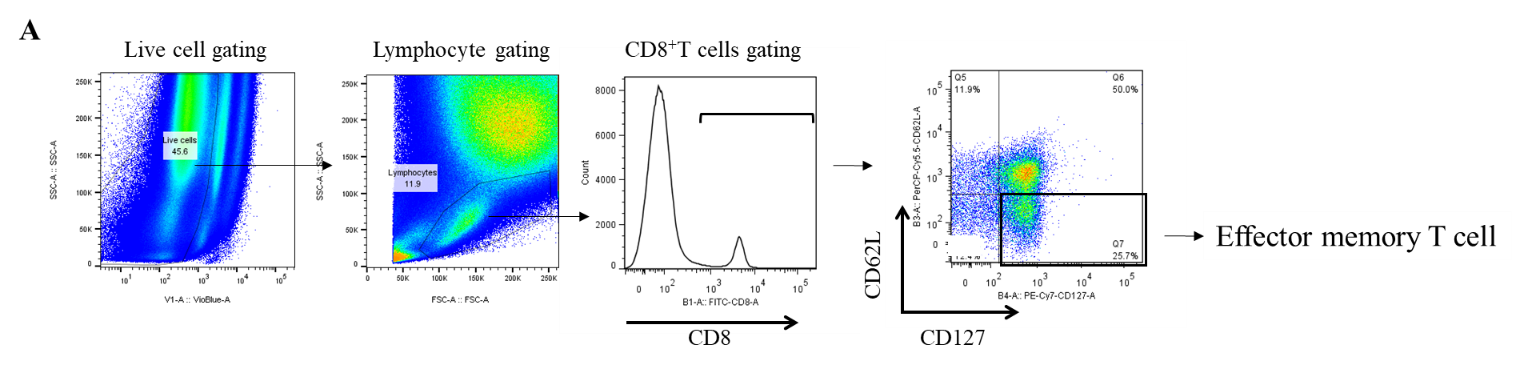


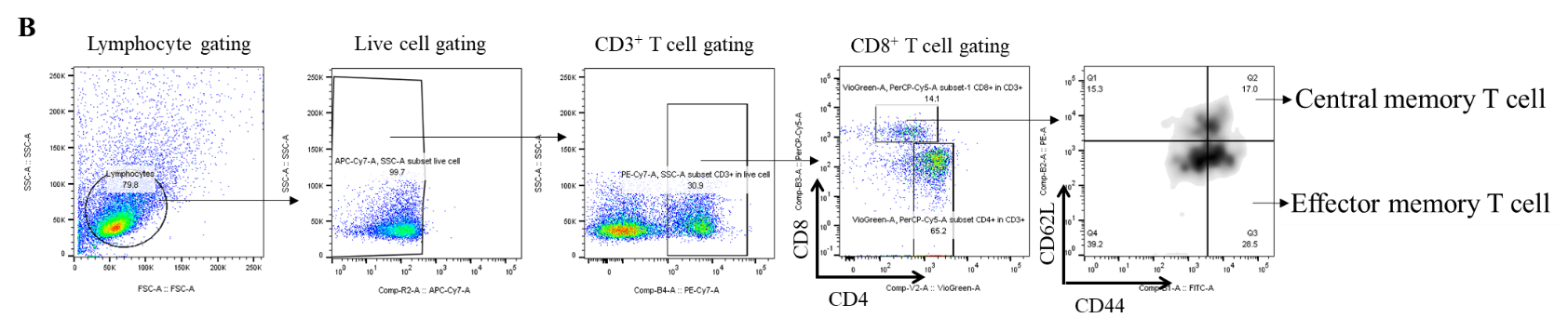


**Supplementary Figure 4.** **A.** Gating strategy for analyzing effector memory T cell in TIL in Fig 4D, 4E and Fig. 5C. **B.** Gating strategy for analyzing effector memory T cell in TIL in Fig 4G

## Supplementary Figure 5.


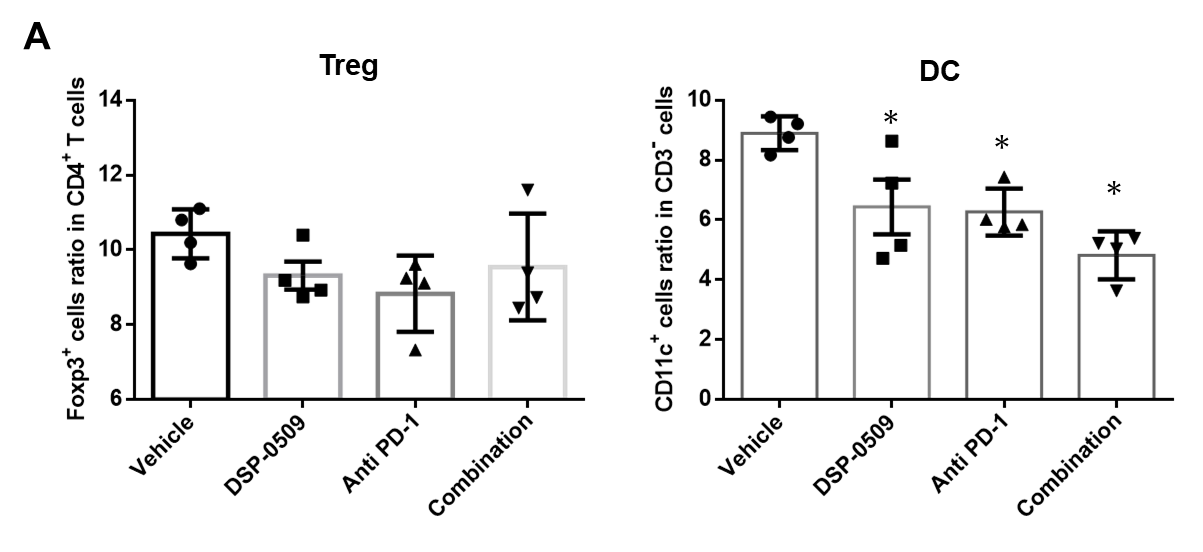


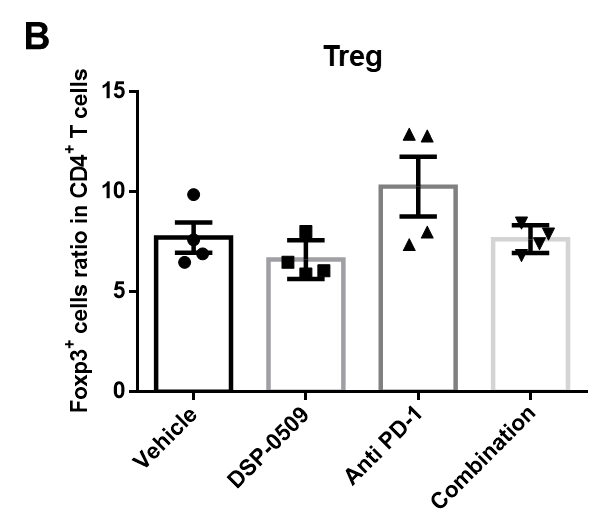


Supplementary Figure 5. Immune profiling in PBMC and lymph node in combination of DSP-0509 with anti-PD-1 antibody in CT26 model. A. Treg and DC ratio in PBMC after DSP-0509 and/or anti-PD-1 antibody treatment. Whole blood was collected at 2 h after second dose of DSP-0509 in CT26 model. B. Treg ratio in lymph node after DSP-0509 and/or anti-PD-1 antibody treatment. Axillary lymph node was collected at 2 h after second dose of DSP-0509 in CT26 model.

## Supplementary Figure 6.


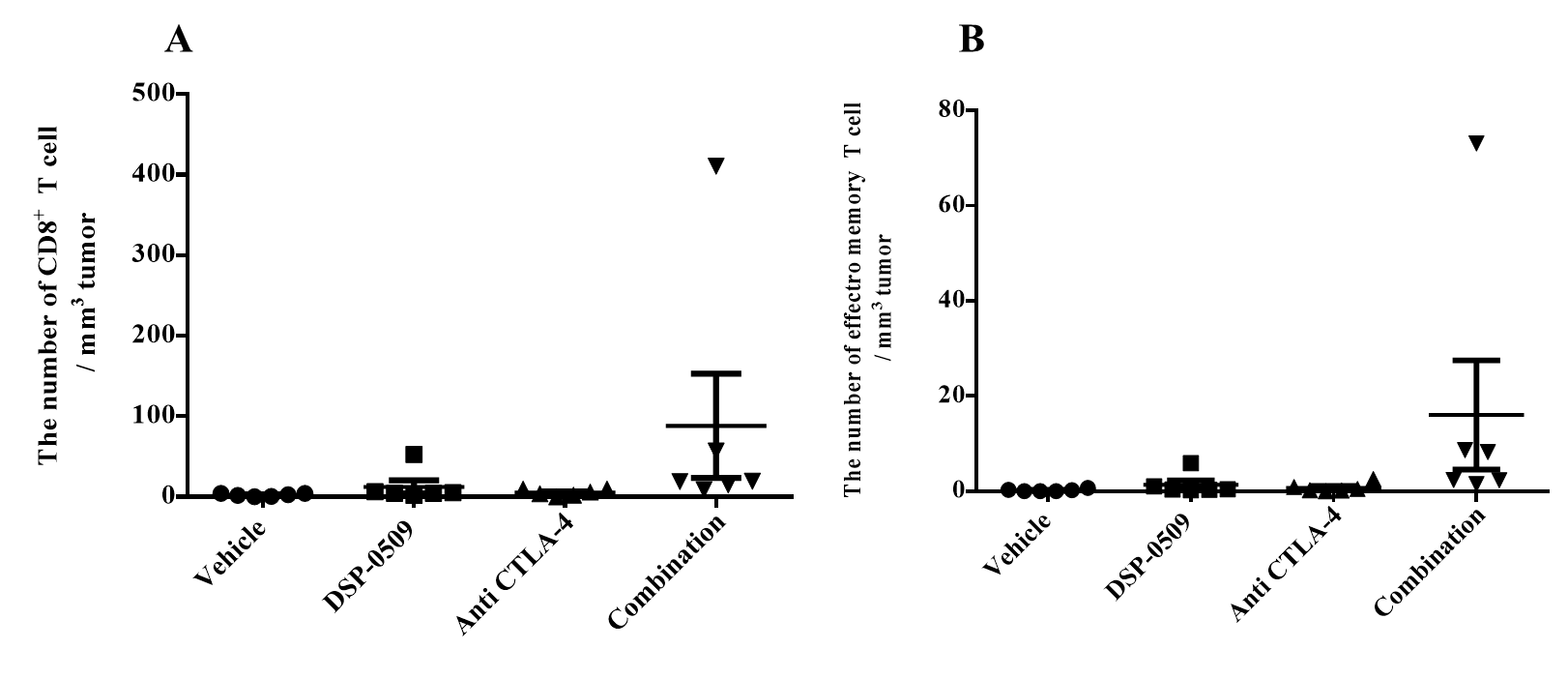


- **Supplementary Figure 6.** Infiltration of CD8^+^ T cell and effector memory T cell in CT26 tumor treated with DSP-0509 and/or anti CTLA-4 antibody. **A.** The number of CD8^+^ T cell in CT26 tumor. **B.** The number of effector memory T cell in CT26 tumor.

## Supplementary Figure 7.


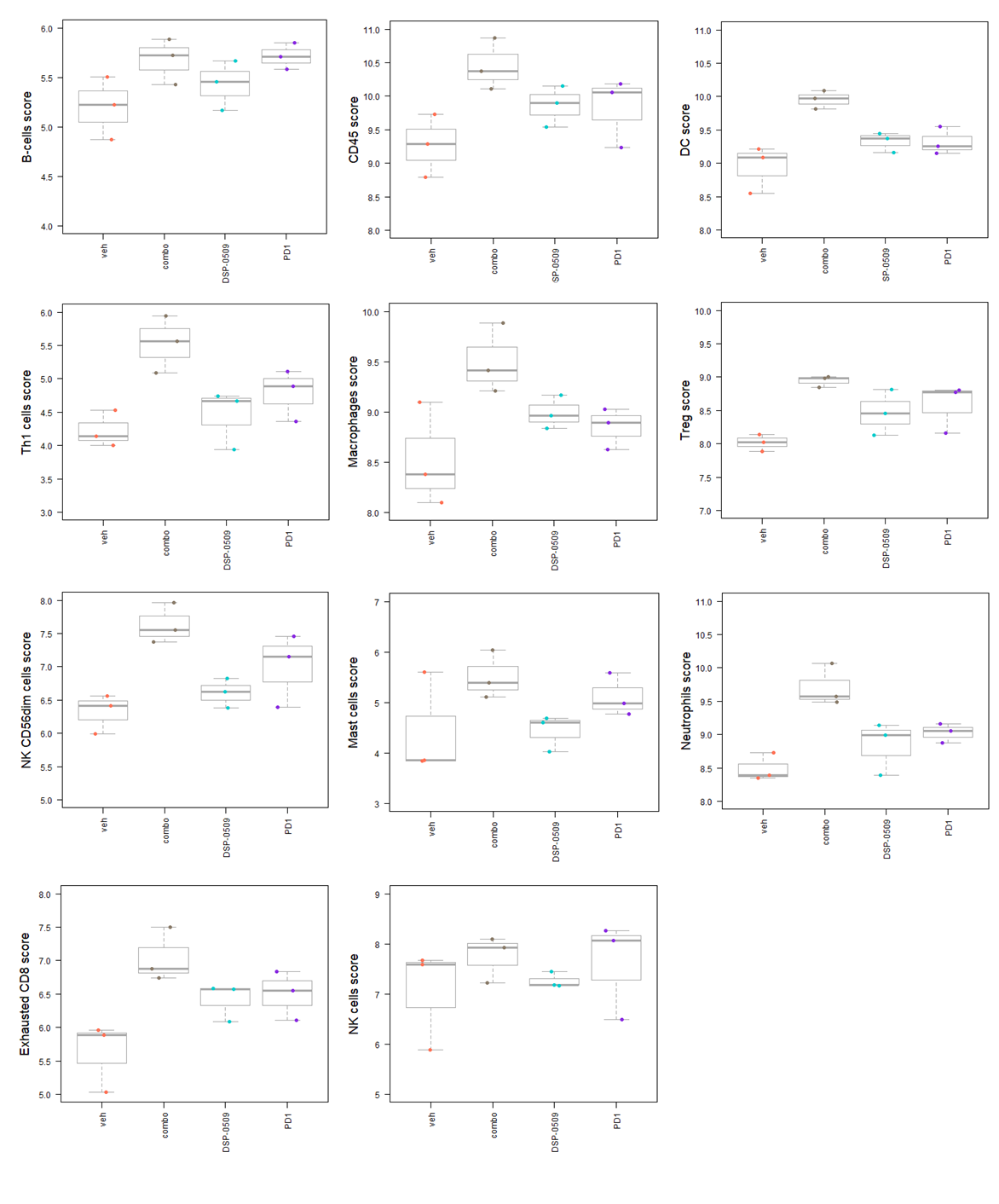


- **Supplementary Figure 7.** Cell type profiling score based on mRNA expression. mRNA was extracted from CT26 tumor treated with DSP-0509 and/or anti PD-1 antibody. Cell type score was calculated using nSolver advanced package.

## Supplementary Figure 8.
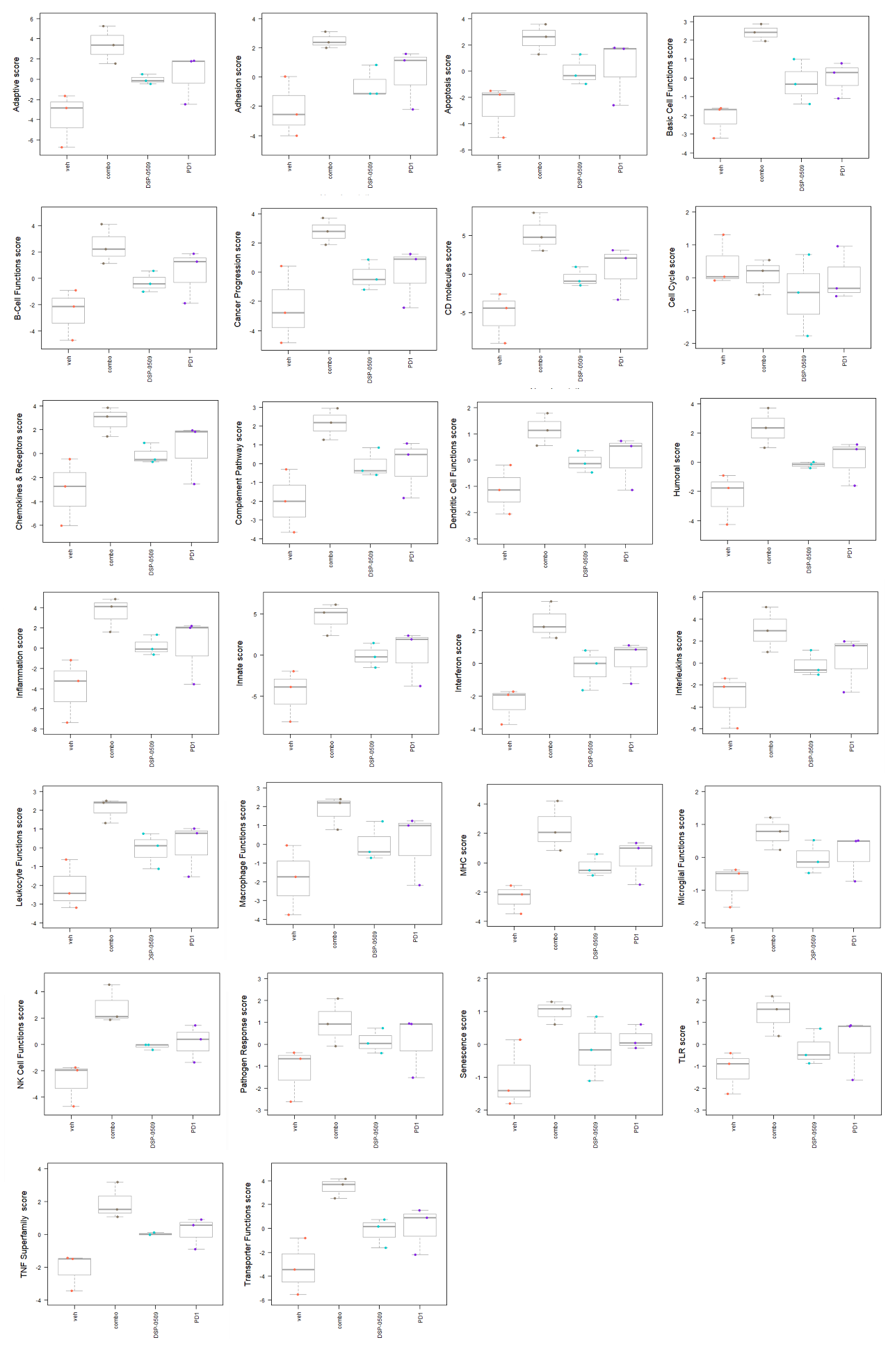


- **Supplementary Figure 8.** Pathway score analysis based on mRNA expression. mRNA was extracted from CT26 tumor treated with DSP-0509 and/or anti PD-1 antibody. Pathway score was calculated using nSolver advanced package.
